# Challenging Postural Tasks Increase Asymmetry in Patients with Parkinson’s Disease

**DOI:** 10.1371/journal.pone.0137722

**Published:** 2015-09-14

**Authors:** Victor Spiandor Beretta, Lilian Teresa Bucken Gobbi, Ellen Lirani-Silva, Lucas Simieli, Diego Orcioli-Silva, Fabio Augusto Barbieri

**Affiliations:** 1 Univ Estadual Paulista, UNESP, Posture and Gait Studies Laboratory, Department of Physical Education, Bioscience Institute, Rio Claro, São Paulo, Brazil; 2 Univ Estadual Paulista, UNESP, Laboratory of Information, Vision and Action, Department of Physical Education, Faculty of Science, Bauru, São Paulo, Brazil; Oslo University Hospital, NORWAY

## Abstract

The unilateral predominance of Parkinson’s disease (PD) symptoms suggests that balance control could be asymmetrical during static tasks. Although studies have shown that balance control asymmetries exist in patients with PD, these analyses were performed using only simple bipedal standing tasks. Challenging postural tasks, such as unipedal or tandem standing, could exacerbate balance control asymmetries. To address this, we studied the impact of challenging standing tasks on postural control asymmetry in patients with PD. Twenty patients with PD and twenty neurologically healthy individuals (control group) participated in this study. Participants performed three 30s trials for each postural task: bipedal, tandem adapted and unipedal standing. The center of pressure parameter was calculated for both limbs in each of these conditions, and the asymmetry between limbs was assessed using the symmetric index. A significant effect of condition was observed, with unipedal standing and tandem standing showing greater asymmetry than bipedal standing for the mediolateral root mean square (RMS) and area of sway parameters, respectively. In addition, a group*condition interaction indicated that, only for patients with PD, the unipedal condition showed greater asymmetry in the mediolateral RMS and area of sway than the bipedal condition and the tandem condition showed greater asymmetry in the area of sway than the bipedal condition. Patients with PD exhibited greater asymmetry while performing tasks requiring postural control when compared to neurologically healthy individuals, especially for challenging tasks such as tandem and unipedal standing.

## Introduction

More than half of patients with Parkinson’s disease (PD) exhibit unilateral motor impairments [1–3]. This unilateral predominance may persist throughout the span of disease progression (for 20–30 years) [4]. However, most patients present bilateral impairments later on in the disease [4]. One possibility for the unilateral predominance of disease symptoms seems to be the differences in striatal uptake between the hemispheres in both the caudate and putamen nuclei [5–6]. In addition, the dominant cerebral hemisphere has an extensive distribution throughout the cortical-basal ganglia-thalamic circuitry [7–8]. The asymmetrical nature of PD affects both the upper and lower limbs and seems also to affect balance control during static tasks [9–10].

Previous studies have revealed that patients with PD demonstrate greater postural control asymmetry in a bipedal standing condition than neurologically healthy individuals, although not all patients show balance control asymmetries [9–13]. However, these findings resulted from only simple bipedal standing tasks. Challenging postural tasks, such as unipedal and tandem standing, that are required for daily life activities, can exacerbate the postural adjustments of the body [14]. Although patients with PD do have difficulties with maintaining balance during static situations, they are especially impaired when they have to modulate their behavior [15], which is required in challenging standing tasks. Therefore, these tasks may increase the use of the least affected limb, which could demonstrate the effective use of this leg during postural control and raise the question as to whether the less affected side in patients with PD could compensate for the more affected side. However, it is necessary to be careful since compensation cannot be investigated on unipedal standing. In addition, understanding the underlying factors that may be contributing to abnormal performance in balance, such as asymmetry and challenging tasks, could provide insight into why patients with PD have a high risk of falling.

The aim of this study was to analyze the impact of challenging standing tasks on postural control asymmetry in patients with PD and neurologically healthy individuals (i.e., the control group). Towards this end, asymmetry was evaluated in subjects performing standing tasks that require different levels of difficulty (bipedal, tandem and unipedal standing). This study sought to test the following hypotheses: i) postural control asymmetry would be higher in patients with PD than in the control group regardless of the specific task examined; ii) postural control asymmetry would be higher during the performance of challenging tasks (i.e., individuals performing unipedal or tandem standing) compared to easier tasks (i.e., bipedal standing), especially in patients with PD.

## Methods

Patients diagnosed with idiopathic PD were selected from a specialized PD center in São Paulo–Brazil. Twenty patients with PD (ten men) and twenty neurologically healthy individuals (control group) participated in this study (Table 1). The individuals with PD were referred to the current study by local neurologists, and their PD diagnoses were confirmed by expert neurologists associated with our group according to the defined UK Brain Bank Criteria [16]. The groups were matched according to their sex, age, weight, height and cognitive level.

**Table 1:** Means and standard deviations of the anthropometric and clinical data of patients with PD (PD) and the control group (CG).

	Age (years)	Weight (kg)	Height (m)	MMSE (pts)	H&Y (score)	UPDRS motor (score)
**CG**	69.35±6.38	70.53±11.11	1.62±0.08	29.00±1.49	————	——————
**PD**	72.30±5.80	71.43±5.57	1.62±0.07	28.30±1.81	1.92±0.29	22.3±7.42

MMSE- Mini Mental State Examination

H&Y–Hoehn & Yahr scale

UPDRS—Unified Parkinson’s Disease Rating Scale.

The following exclusion criteria were used: under 60 years of age (PD is more prevalent over 60 years of age [17–18] and young-onset PD–from 20 to 50 years old—presents different genetic aspects and disease progression than older people with PD [19]); a Hoehn & Yahr Scale [20–21] score above three (people with PD above stage 3 present disabling motor deficiencies and very different postural patterns than the previous stages, with, sometimes, loss of posture independence, which could affect our study); cognitive decline; and musculoskeletal, orthopedic and/or visual impairments that prevent the subject from performing the required tasks. In addition, the inclusion criterion was that the patients with PD had to be taking PD medication. We chose to analyze the patients in an ON state because we wanted to examine the influence of challenging postural tasks with the patient in a position of better motor control. In addition, the patient spends the majority of the day under the effects of medication. Therefore, the results with patients in ON state could be more clinically relevant. Thus, we preferred to analyze the patients in conditions, which were as similar as possible to their daily behavior. If a patient is evaluated in the OFF state, the asymmetry could be exacerbated due to motor and non-motor fluctuations that patients presents in this state, and this may not represent the patient’s daily characteristics. Finally, none patients presents orthopedic conditions, such as artificial joints and/or herniated discs/stenosis.

The study was approved by the research ethics committee of the São Paulo State University at Rio Claro–Brazil (#0227/2013). The participants provided written informed consent to participate in the clinical and postural evaluations. The patients with PD performed all the assessments while in an optimal medicated state (approximately one hour after their last dose of medication).

First, an expert neuropsychiatrist performed an anamneses to characterize the PD and control groups, especially the side on which the symptoms were occurring. Next, the patients were evaluated clinically to determine the stage of PD, using the H&Y scale and the motor portion of the Unified Parkinson's Disease Rating Scale–UPDRS [22]. In addition, cognitive impairments in both groups were analyzed using the Mini Mental State Examination [23–24].

Footedness was assessed in the control group by asking all participants to stand on one leg [25]. The limb that each individual initially chose to stand on was considered as the preferred limb. For patients with PD, motor UPDRS items 20–23 and 25–26 were used to assess appendicular asymmetry [2] (see Table A in S1 Dataset). The most severely affected limb was determined by finding the difference between the scores for the right and left limbs in the aforementioned UPDRS items. When this calculation resulted in a positive value, the right limb was considered to be the more severely affected limb, but when negative values were obtained, this indicated that the left limb was more severely affected.

Two separate force plates– 50x50 cm (AccuGait, Advanced Mechanical Technologies, Boston, MA) were used to analyze postural control with a frequency of 200 samples/s. The force plates were positioned at a distance of 5 cm from each other. Participants performed each of the following three tasks. Firstly, for bipedal standing, the participants positioned one foot on each force plate and were required to stand quietly in an upright position. Their feet were positioned in parallel to each other, and they were placed at a similar distance from the pelvis. Participants performed three 30 s trials of bipedal standing. Secondly, for tandem adapted standing, participants positioned one foot on each force plate, but one foot was placed in front of the other. Participants performed three 30 s trials with each lower limb positioned in front. Thirdly, for unipedal standing, the participants used only one leg to stand. Participants performed three 30 s trials with each limb for unipedal standing. Due to the difficulties that many of the participants experienced when trying to stand on one foot, especially patients with PD, all participants were allowed to touch a wooden support as lightly as possible. All participants used this support during the unipedal standing trials. For each of the three tasks, the location of the participants’ feet was recorded on a sheet to allow each participant to maintain the same foot position for each trial.

The order of each block of trials was bipedal standing first (condition a), tandem adapted standing second (condition b) and unipedal standing last (condition c). In conditions b and c, whether the right or left foot was placed in front or used for unipedal standing was randomized for each participant. For each of the three conditions, participants were instructed to stand quietly with their gaze directed at a target that was positioned at eye level 1 m away from the participant.

The three force and moment components were acquired in the vertical, anterior-posterior and mediolateral directions. The first 10 s of each recording was ignored systematically to avoid potential disturbances resulting from delayed stabilization after the participant stepped onto the force plates of recording equipment [26]. The mean center of pressure (CoP) signal was also calculated for each analysis. The data were then filtered with a fourth order low-pass Butterworth filter with a cut-off frequency (10 Hz) determined by residual analysis [26].

For the bipedal and tandem standing, the resultant CoP signal of the ground reaction forces was determined in a 2-dimensional transverse plane by means of digital moment-of-force calculations for each force plate [9–10]. For unipedal standing, the CoP was calculated in a similar manner as for the other conditions, but for only one force plate. In the tandem standing condition, the CoP of the limb positioned behind was used for the analysis. The following parameters in the anterior-posterior and mediolateral directions were analyzed for each force plate: the mean velocity of sway (i.e., the displacement of the total sway of the CoP divided by the total duration of the trial); and the root mean square (RMS) of sway displacement (i.e., the CoP variability around the mean CoP trajectory). In addition, the area of sway (i.e., the area of an ellipse that contains 95% of the CoP data) and the total displacement of sway (i.e., the length of the CoP trajectory on the support base) was calculated. We choose to use these parameters because they indicate global analysis, which numerically expresses the “size” of the sway patterns and each parameter provides one piece of information on postural control, for example, total displacement indicates how much the individual oscillates and RMS indicates the variability of the system [27]. In addition, we chose the classic posturographic parameters, which are most commonly found in the literature and the analysis is based on the underlying assumption that the CoP signal represents actual movement [27].

The asymmetry between the most affected and the least affected limbs for patients with PD and the preferred and the non-preferred limbs for the control group was analyzed using a symmetric index–SI [28–29]:

$$SI\;=\;\left(\frac{value\;of\;LA\;or\;D\;limb-value\;of\;MA\;or\;ND\;limb}{value\;of\;LA\;or\;D\;limb+value\;of\;MA\;or\;ND\;limb}\right)X\;100\%$$

Where MA is the most affected limb, D is the dominant limb, LA is the least affected limb and ND is the non-dominant limb. A value of zero for index indicates that there is no difference between sides.

To calculate the symmetric index, first we calculated the average of each CoP parameter for the dominant and non-dominant, or most and least affected, limb for each participant. Next, the symmetry index for each participant was calculated according to postural conditions. Statistical analyses were performed using SPSS software (version 18.0) for Windows (p<0.05). The data were normally distributed and the assumption of sphericity was not violated, as verified by the Shapiro–Wilk and Mauchly tests, respectively. The symmetric index (absolute values) of the CoP parameters were analyzed using two-way ANOVAs (group: patients with PD and the control group X condition: bipedal, tandem and unipedal standing). When the ANOVA noted significant differences, Tukey’s post hoc tests were performed.

## Results

In S1 Dataset, the anthropometric and clinical characteristics are presented, in addition to the symmetry index of CoP parameters in the bipedal, tandem and unipedal conditions for each individual of both groups: PD and control group (see Individual S1 Dataset). The ANOVAs revealed significant main effects of group (F_5,34_ = 4.99, p<0.001) and condition (F_10,29_ = 3.79, p<0.02) as well as a significant group*condition interaction (F_10,29_ = 1.98., p<0.02). For group (Table 2), post hoc tests revealed that patients with PD presented greater asymmetry of the mediolateral RMS (p<0.03) and area of sway (p<0.01) than the control group. For condition (Table 2), greater asymmetry was observed for the anterior-posterior RMS in the bipedal condition when compared to the tandem condition (p<0.03). For the area of sway analysis, the tandem condition presented greater asymmetry than the bipedal condition, and for the mediolateral RMS analysis, the unipedal condition exhibited greater asymmetry than the bipedal condition (p<0.02).

**Table 2:** Means and standard deviations of the CoP parameters symmetric index (%) in patients with PD (PD) and the control group (CG).

Parameter (%)		Effects of group	Bipedal	Tandem	Unipedal
Total displacement of	**CG**	12.01±1.67	12.28±1.33	12.51±1.48	11.25±1.16
sway	**PD**	12.38±1.50	12.22±1.92	12.94±2.01	11.96±1.80
**Effects of task**			**12.26±1.63**	**12.72±1.53**	**11.60±1.36**
Anterior-posterior	**GC**	12.96±1.68	8.93±0.70	12.79±1.56	17.16±1.86
mean velocity of sway	**PD**	16.19±2.41	10.95±1.78	18.38±3.06	28.25±3.26
**Effects of task**			**9.94±1.10** ^**#**^ ^**&**^	**15.58±2.06**	**22.70±2.86**
Mediolateral mean	**CG**	12.93±1.48	12.60±1.22	12.31±2.02	13.88±1.13
velocity of sway	**PD**	14.01±2.01	16.33±2.79	14.39±2.16	11.31±1.60
**Effects of task**			**14.46±2.05**	**13.34±2.10**	**12.59±1.43**
Anterior-posterior	**CG**	15.74±3.36	19.61±1.90	14.11±1.82	13.52±3.50
RMS	**PD**	16.00±2.37	16.85±1.47	13.32±1.70	17.84±2.09
**Effects of task**			**18.22±1.77** ^**#**^	**13.71±1.77**	**15.67±2.39**
Mediolateral RMS	**CG**	12.66±1.24*	12.36±1.06	14.03±1.10	11.60±0.92
	**PD**	15.70±3.28	12.42±2.74	15.17±3.77	19.52±3.73
**Effects of task**			**12.38±1.71** ^**&**^	**14.59±1.83**	**15.56±1.77**
Area of sway	**CG**	17.27±1.41*	17.11±1.30	18.76±1.51	15.95±1.15
	**PD**	25.84±4.17	21.03±4.52	28.08±3.66	28.42±4.90
**Effects of task**			**19.06±2.40** ^**#**^	**23.42±2.60**	**22.18±2.90**

* indicates a significant difference between the PD and CG.

# indicates a significant difference between the bipedal and tandem conditions.

&indicates a significant difference between the bipedal and unipedal conditions.

**Fig 1:**
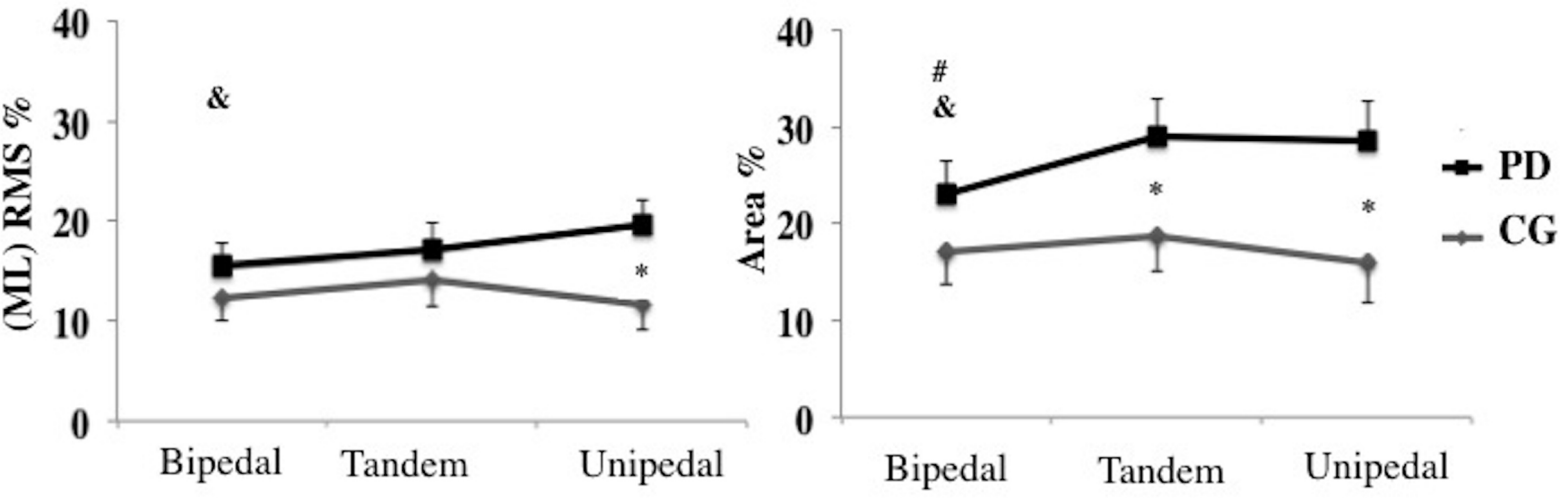
Group*condition interactions for mediolateral (ML) RMS and area of body sway. * indicates a significant difference between the PD and CG; # indicates a significant difference between the bipedal and tandem conditions; & indicates a significant difference between the bipedal and unipedal conditions.

Concerning group*condition interaction (Fig 1), patients with PD showed greater symmetric index for mediolateral RMS in the unipedal condition (p<0.002) and for area of sway in the tandem and unipedal conditions than the control group. In addition, only for patients with PD, the unipedal condition showed greater asymmetry in the mediolateral RMS and area of sway than the bipedal condition (p<0.001 and p<0.002, respectively) and the tandem condition showed greater asymmetry in the area of sway than the bipedal condition (p<0.002).

## Discussion

Although asymmetries in postural control have been previously reported in patients with PD performing bipedal quiet standing tasks [9–13], this is the first study to investigate the impact of challenging postural tasks, such as tandem and unipedal standing, on postural control asymmetry in patients with PD. The main finding of this study was that patients with PD exhibit greater asymmetry in postural control while performing more challenging tasks (i.e., the tandem and unipedal conditions) when compared to easier tasks (i.e., the bipedal condition). In contrast, this effect was not observed in neurologically healthy individuals. Thus, our hypothesis that patients with PD would exhibit greater asymmetry of postural control than the control group was confirmed.

The group*condition interactions indicated that the asymmetry differences between the groups occurred only during the two most challenging tasks. The greater asymmetry for anterior-posterior RMS in the bipedal condition compared to the tandem condition was contrary to our hypothesis. Therefore, our second hypothesis that increasing the difficulty of the tasks would only influence the asymmetry of postural control in individuals with PD was partially confirmed. The asymmetry difference between conditions (bipedal and tandem conditions) may be explained by the differences in foot position between conditions. In the bipedal condition mediolateral balance is improved due to a large basis of support in this direction, while in the tandem condition the anterior-posterior balance is improved. Therefore, individuals adjust their posture in the anterior-posterior direction during the bipedal condition, which demonstrates greater flexibility in this condition. Given that we analyzed older people and people with PD, who display higher postural asymmetry due to the effects of aging and disease [30–32], the increased adjustments in this direction revealed greater asymmetries during the bipedal condition. Based on the results observed, we will present two lines of reasoning in the following paragraphs. Firstly, we will discuss explanations for the postural control asymmetry in people with PD. Secondly, we will offer interpretations in terms of greater postural control asymmetry during challenging postural tasks in patients with PD. It is important to remember that the individuals with PD were analyzed under PD medication effects. Maybe an investigation without medication effects could present different findings.

### 4.1. Postural control asymmetry in people with PD

Individuals with PD exhibited greater asymmetry in postural control when compared to neurologically healthy individuals. The findings of this study corroborate previous research that has also reported greater asymmetry in patients with PD performing bipedal postural tasks [9–13]. However, our values for the symmetry index of the anterior-posterior velocity of sway were lower values to previous studies [9]. Two aspects possibly contributed to this difference: a) stage of disease: almost half (n = 9) of the participants in the current study had unilateral PD while only 4 individuals (number total of participants = 17) in the previous study were in this stage of the disease; b) symmetry index calculation: Geurts and collaborators [9] used the factor 2 in the equation of the symmetry index, while we did not use this factor in our symmetry index calculation. This factor considers the asymmetry from -200 to 200%.

The asymmetry in postural control observed in patients with PD may be interpreted as reflecting asymmetric degeneration of dopaminergic neurons in the substantia nigra [4]. This causes impaired adjustments in postural responses and difficulty in adjusting to task context [31]. In addition, asymmetry in postural control could indicate a compensation strategy if one limb shows greater balance impairments than the other during bipedal and tandem stance [13,32–33]. Although prior studies have reported that compensation strategies can be desirable and effective in maintaining balance [13,33], these strategies can mask the extent of the deficiency in the more severely affected limb and may result in falls, especially while patients are performing tasks that require greater balance. On the other hand, the increased contribution of the least affected limb might be interpreted as a compensation to avoid more impaired balance control, decreasing the use of the most affected limb during postural control.

### 4.2. Effects of challenging postural tasks on asymmetry

Challenging postural tasks increase the asymmetry of postural control in patients with PD. The main explanation of this asymmetrical behavior seems to be the lack of a compensatory strategy in challenging postural tasks, specially during one leg stance that one leg is not contact with the ground, which prevents compensation between the legs. Bipedal tasks with less postural requirement, such as bipedal quiet stance, facilitate the use of least affected limb to control posture [13,32–33]. In addition, the following aspects may explain more asymmetry in challenging postural tasks: i) asymmetric nigral dopaminergic neuron cell death, i.e., in one hemisphere more cells die compared to the ohter (as mentioned above) [5–6], as well as asymmetry in the cells distribution throughout the cortical-basal ganglia-thalamic circuitry [7–8], which seems to indicate a control problem instead of a mechanical issue. This statement is supported by Rocchi and colleagues [10] that suggest that bilateral stimulation in the globus pallidus or subthalamic nucleus can improve symmetry of postural control between the legs in people with PD; ii) one side is affected first in PD and this motor asymmetry is preserved through disease progression [4], which seems to evidence an increased use of the least affected side to perform the tasks, mainly easier postural tasks [12,13]. Thus, when the people with PD are required to use effectively their most affected limb to control the balance during challenging postural task, they show an increase in the asymmetry differently to neurological healthy people that did have (or have less) no asymmetrical effects. To conclude our rationale, we could suggest that many of falls of people with PD seems to happen due to use of most affected limb in the tasks. Most of falls (~30%) in patients with PD happens during complex tasks, such as obstacle crossing [34], which requires the use of most affected limb and inhibits an appropriate adjustment in these challenging conditions.

Patients with PD demonstrate a higher degree of body sway while performing complex postural tasks [30]. Previous studies suggest that the effort of static postural control is minimal around the natural feet distance (bipedal condition) [35], which seems to reduce the asymmetry in this condition compared to challenging postures (narrow bases), such as tandem and unipedal conditions. Narrow stance increases postural deficits since postural responses require large lateral flexion of the trunk (to most or least affected side) when the legs are close together [36–37], which is impaired in people with PD [32,11]. In addition, patients with PD have difficulty adapting the magnitude and patterns of postural responses according to changes in postural demand, which may be explained by the importance of basal ganglia to deal with biomechanical constraints related to challenging postural tasks [31]. In addition, the quiet posture is controlled using a closed-loop system, which is characterized by feedback control that continuously adjusts the posture according to somatosensory information [38]. However, patients with PD have sensory and perceptual deficits [10,36], which during challenging postural tasks seem to exacerbate the mechanical adjustments of the body and present postural control asymmetry. A previous study that applied external continuous perturbations during the performance of a postural task have suggested an increase in asymmetry in patients with PD, but it was not conclusive since the authors assessed the patients in an OFF medication state [12–13].

The greater asymmetry observed in the mediolateral RMS (difference between the bipedal and unipedal conditions) and area of sway (difference between the bipedal and tandem conditions) reflects the ineffectiveness of postural control and deficits in fine tuning movements, which may be related to the poor use of somatosensory information by patients with PD in mediolateral and anterior-posterior asymmetry [10,39]. The greatest asymmetry observed in the mediolateral direction may indicate that increased effort is required to maintain balance in this direction [40], which has been reported to be impaired in patients with PD [30,33]. This finding may also be related to the decrease in trunk coordination that has been reported in this population [32,11]. On the other hand, we cannot forget that trunk coordination is also impaired in the sagittal plane [32,11], which may influence the asymmetry in the area of sway. The high levels of asymmetry in the area of sway can cause deficits in stability limits, which could be related to falls [41]. This finding seems to be an indication of increased contribution of the most affected limb in the tandem condition (see S1 Dataset). In addition, these findings seem to confirm that asymmetry and challenging tasks influence postural control and these changes could result in an increased likelihood of falling [42]. Therefore, new studies should be performed to investigate the relationship between falling patients, asymmetry and the influence of physical activity.

## Conclusions

In conclusion, patients with PD exhibit greater asymmetry in postural control than neurologically healthy individuals, especially while performing challenging tasks such as tandem and unipedal tasks. Because of this, we recommend using challenging postural tasks to study postural asymmetry in PD.

## Supporting Information

S1 Dataset Anthropometric and clinical characteristics, and mean raw data and symmetry index of CoP parameters in the bipedal, tandem and unipedal condition for each individual. **Table A.** Anthropometric and clinical characteristics for each patient with PD and each neurological healthy individual. Part.–participant; MMSE- Mini Mental State Examination; HY–Hoehn &Yahr scale; UPDRS—Unified Parkinson’s Disease Rating Scale–motor part; Limb–preferred limb (R–right; L–left); SI–symmetric index (calculated through motor UPDRS items 20–23 and 25–26). Positive value indicates that the most affected side is the right and negative value indicates that the most affected side is the left. **Table B.** Symmetry index (%) of CoP parameters for each patient with PD in bipedal, tandem and unipedal conditions. Total disp.–total displacement of sway; vel. of sway–mean velocity of sway; AP–anterior-posterior; ML–mediolateral. **Table C.** Symmetry index (%) of CoP parameters symmetric index for each neurological healthy individual in bipedal, tandem and unipedal conditions. Total disp.–total displacement of sway; vel. of sway–mean velocity of sway; AP–anterior-posterior; ML–mediolateral. **Table D.** Mean raw data of least affected limb for each patient with PD in bipedal, tandem and unipedal conditions. Total disp.–total displacement of sway; vel. of sway–mean velocity of sway; AP–anterior-posterior; ML–mediolateral. **Table E.** Mean raw data of most affected limb for each patient with PD in bipedal, tandem and unipedal conditions. Total disp.–total displacement of sway; vel. of sway–mean velocity of sway; AP–anterior-posterior; ML–mediolateral. **Table F.** Mean raw data of dominant limb for each neurological healthy individual in bipedal, tandem and unipedal conditions. Total disp.–total displacement of sway; vel. of sway–mean velocity of sway; AP–anterior-posterior; ML–mediolateral. **Table G.** Mean raw data of non-dominant limb for each neurological healthy individual in bipedal, tandem and unipedal conditions. Total disp.–total displacement of sway; vel. of sway–mean velocity of sway; AP–anterior-posterior; ML–mediolateral.
(DOCX)
