# Supplementary material for: Challenging Postural Tasks Increase Asymmetry in Patients with Parkinson’s Disease
Source: PLoS One. 2015 Sep 14;10(9):e0137722. doi: 10.1371/journal.pone.0137722 (PMC4569579; doi:10.1371/journal.pone.0137722)
Supplement: S1 Dataset — Table A. Anthropometric and clinical characteristics for each patient with PD and each neurological healthy individual. Part.–participant; MMSE- Mini Mental State Examination; HY–Hoehn &Yahr scale; UPDRS—Unified Parkinson’s Disease Rating Scale–motor part; Limb–preferred limb (R–right; L–left); SI–symmetric index (calculated through motor UPDRS items 20–23 and 25–26). Positive value indicates that the most affected side is the right and negative value indicates that the most affected side is the left. Table B. Symmetry index (%) of CoP parameters for each patient with PD in bipedal, tandem and unipedal conditions. Total disp.–total displacement of sway; vel. of sway–mean velocity of sway; AP–anterior-posterior; ML–mediolateral. Table C. Symmetry index (%) of CoP parameters symmetric index for each neurological healthy individual in bipedal, tandem and unipedal conditions. Total disp.–total displacement of sway; vel. of sway–mean velocity of sway; AP–anterior-posterior; ML–mediolateral. Table D. Mean raw data of least affected limb for each patient with PD in bipedal, tandem and unipedal conditions. Total disp.–total displacement of sway; vel. of sway–mean velocity of sway; AP–anterior-posterior; ML–mediolateral. Table E. Mean raw data of most affected limb for each patient with PD in bipedal, tandem and unipedal conditions. Total disp.–total displacement of sway; vel. of sway–mean velocity of sway; AP–anterior-posterior; ML–mediolateral. Table F. Mean raw data of dominant limb for each neurological healthy individual in bipedal, tandem and unipedal conditions. Total disp.–total displacement of sway; vel. of sway–mean velocity of sway; AP–anterior-posterior; ML–mediolateral. Table G. Mean raw data of non-dominant limb for each neurological healthy individual in bipedal, tandem and unipedal conditions. Total disp.–total displacement of sway; vel. of sway–mean velocity of sway; AP–anterior-posterior; ML–mediolateral. (DOCX) [file pone.0137722.s001.docx]

**Anthropometric and clinical characteristics, and mean raw data and symmetry index of CoP parameters in bipedal, tandem and unipedal condition for each individual**

**Table A.** Anthropometric and clinical characteristics for each patient with PD and each neurological healthy individual. Part. – participant; MMSE- Mini Mental State Examination; HY – Hoehn &Yahr scale; UPDRS - Unified Parkinson’s Disease Rating Scale – motor part; Limb – preferred limb (R – right; L – left); SI – symmetric index (calculated through motor UPDRS items 20-23 and 25-26). Positive value indicates that the most affected side is the right and negative value indicates that the most affected side is the left.

| **Control group** | | | | | |  |  | **PD group** | | | | | | | | |
| --- | --- | --- | --- | --- | --- | --- | --- | --- | --- | --- | --- | --- | --- | --- | --- | --- |
| **Part.** | **Gender** | **Age** | **Weight** | **Height** | **MMSE** | **Limb** |  | **Part.** | **Gender** | **Age** | **Weight** | **Height** | **MMSE** | **HY** | **UPDRS** | **SI** |
|  | **(M/F)** | **(years)** | **(kg)** | **(m)** | **(pts)** |  |  |  | **(M/F)** | **(years)** | **(kg)** | **(m)** | **(pts)** | **(score)** | **(score)** | **(score)** |
| **1** | M | 80 | 79.7 | 1.67 | 29 | R |  | **1** | M | 81 | 78 | 1.67 | 29 | 2 | 28 | -3 |
| **2** | M | 80 | 55.3 | 1.65 | 30 | R |  | **2** | M | 78 | 74.7 | 1.63 | 27 | 2.5 | 40 | -3 |
| **3** | F | 75 | 70.7 | 1.52 | 28 | R |  | **3** | F | 75 | 67.7 | 1.54 | 24 | 1.5 | 15 | -1 |
| **4** | F | 69 | 64 | 1.65 | 27 | R |  | **4** | F | 69 | 68 | 1.46 | 28 | 2 | 41 | -5 |
| **5** | F | 58 | 70.9 | 1.64 | 30 | R |  | **5** | F | 61 | 64.5 | 1.51 | 30 | 2 | 17 | -3 |
| **6** | F | 69 | 67.25 | 1.5 | 30 | R |  | **6** | F | 70 | 62.5 | 1.57 | 25 | 2 | 22 | -4 |
| **7** | F | 66 | 47.5 | 1.5 | 28 | R |  | **7** | F | 73 | 78.8 | 1.65 | 25 | 2 | 28 | -2 |
| **8** | M | 68 | 91 | 1.73 | 25 | L |  | **8** | M | 68 | 66.2 | 1.59 | 28 | 2 | 21 | -1 |
| **9** | F | 60 | 63.1 | 1.55 | 30 | R |  | **9** | F | 75 | 75.9 | 1.65 | 29 | 2 | 27 | -3 |
| **10** | M | 64 | 71 | 1.72 | 29 | R |  | **10** | M | 64 | 82.9 | 1.71 | 30 | 1.5 | 15 | 4 |
| **11** | F | 64 | 64.8 | 1.54 | 30 | R |  | **11** | F | 77 | 62.8 | 1.56 | 30 | 2.5 | 32 | 4 |
| **12** | F | 68 | 66.5 | 1.65 | 30 | R |  | **12** | F | 79 | 69.5 | 1.59 | 28 | 2 | 27 | -7 |
| **13** | M | 67 | 78.3 | 1.7 | 30 | L |  | **13** | M | 67 | 70 | 1.57 | 30 | 1.5 | 16 | -1 |
| **14** | M | 79 | 84.4 | 1.66 | 29 | R |  | **14** | M | 79 | 72 | 1.69 | 28 | 1.5 | 14 | -3 |
| **15** | F | 64 | 52.3 | 1.61 | 30 | R |  | **15** | F | 75 | 75.5 | 1.6 | 30 | 2 | 20 | 1 |
| **16** | M | 75 | 69.8 | 1.58 | 30 | R |  | **16** | M | 75 | 70.5 | 1.72 | 28 | 1.5 | 13 | -2 |
| **17** | M | 65 | 84.7 | 1.74 | 30 | R |  | **17** | M | 62 | 68.5 | 1.71 | 29 | 1.5 | 10 | 1 |
| **18** | F | 75 | 75 | 1.52 | 26 | R |  | **18** | F | 76 | 78 | 1.55 | 29 | 1.5 | 17 | 1 |
| **19** | M | 68 | 81 | 1.72 | 29 | R |  | **19** | M | 69 | 71 | 1.69 | 29 | 1.5 | 9 | 1 |
| **20** | M | 73 | 73.3 | 1.73 | 30 | R |  | **20** | M | 73 | 71.5 | 1.76 | 30 | 1 | 12 | 2 |

**Table B.** Symmetry index (%) of CoP parameters for each patient with PD in bipedal, tandem and unipedal conditions. Total disp. – total displacement of sway; vel. of sway – mean velocity of sway; AP – anterior-posterior; ML – mediolateral.

|  | **BIPEDAL CONDITION** | | | | | | **TANDEM CONDITION** | | | | | | **UNIPEDAL CONDITION** | | | | | |
| --- | --- | --- | --- | --- | --- | --- | --- | --- | --- | --- | --- | --- | --- | --- | --- | --- | --- | --- |
| **Part.** | **total disp.** | **AP vel. of sway** | **ML vel. of sway** | **AP RMS** | **ML RMS** | **area of sway** | **total disp.** | **AP vel. of sway** | **ML vel. of sway** | **AP RMS** | **ML RMS** | **area of sway** | **total disp.** | **AP vel. of sway** | **ML vel. of sway** | **AP RMS** | **ML RMS** | **area of sway** |
| **1** | -4.15 | 12.94 | 6.19 | -8.92 | -2.58 | 15.91 | 1.44 | 26.02 | 2.87 | -14.32 | 8.85 | 43.92 | -10.16 | 40.76 | 16.20 | -61.36 | 16.74 | -53.54 |
| **2** | 17.49 | 16.03 | 21.90 | 8.35 | 13.95 | -16.85 | 0.75 | 2.04 | 5.26 | -2.45 | 1.69 | 0.12 | 14.88 | 50.86 | 19.36 | 32.46 | -9.38 | 40.66 |
| **3** | -13.37 | 7.09 | 12.18 | -8.23 | -15.77 | -25.28 | -3.81 | 37.52 | 1.99 | -21.18 | 1.51 | -37.38 | -3.22 | 42.29 | 11.40 | -17.54 | 15.83 | -26.33 |
| **4** | 10.72 | 0.83 | 26.45 | -5.16 | 12.40 | 7.12 | 3.56 | 4.97 | 3.09 | -1.92 | 3.19 | -6.92 | -10.59 | 3.71 | 16.62 | -2.93 | -19.98 | -22.67 |
| **5** | -7.18 | 2.98 | 9.29 | 1.15 | -7.80 | -1.55 | -2.25 | 16.73 | 5.92 | 6.99 | -12.61 | 12.80 | 14.73 | 16.61 | 11.04 | -3.11 | 20.98 | -2.36 |
| **6** | 42.05 | 27.22 | 55.35 | 29.23 | 42.91 | 55.78 | 44.20 | 42.92 | 45.01 | 44.77 | 48.01 | 57.84 | -7.16 | 14.35 | 11.54 | -4.18 | -15.79 | 18.57 |
| **7** | 2.51 | 6.21 | 14.60 | -16.37 | 2.50 | 2.54 | -15.08 | 16.90 | 26.57 | -10.66 | -20.15 | -37.82 | 10.11 | 31.26 | 16.82 | 33.79 | -12.67 | 29.76 |
| **8** | -15.41 | 16.62 | 14.13 | -18.30 | -14.45 | -18.62 | -28.69 | 43.35 | 24.47 | -54.79 | -19.60 | -8.22 | 34.16 | 29.35 | 33.33 | 29.32 | 39.50 | 62.75 |
| **9** | -7.28 | 4.61 | 10.79 | -2.36 | -6.34 | 6.00 | 3.71 | 6.52 | 2.37 | 1.37 | 1.75 | 24.84 | 5.41 | 4.11 | 5.96 | 2.18 | 10.41 | 11.97 |
| **10** | -12.88 | 7.54 | 13.18 | -12.70 | -14.29 | -30.20 | 4.99 | 7.02 | 16.40 | -12.80 | 5.20 | -10.62 | -4.50 | 32.48 | 5.56 | -20.66 | 1.01 | -29.83 |
| **11** | -3.18 | 1.24 | 2.81 | 1.97 | -3.66 | -7.35 | 5.35 | 1.76 | 2.02 | 4.14 | 10.95 | 13.50 | 5.00 | 11.59 | 11.95 | 1.73 | 15.51 | 8.13 |
| **12** | 14.84 | 7.35 | 11.87 | 24.89 | 15.67 | 23.92 | 6.73 | 29.43 | 8.04 | 10.16 | -1.40 | -10.02 | 10.09 | 33.51 | 13.36 | 24.94 | -6.25 | 19.46 |
| **13** | -29.67 | 17.39 | 37.30 | 9.10 | -32.15 | -20.65 | -17.31 | 12.58 | 26.62 | -15.91 | -17.34 | 1.44 | 19.20 | 12.75 | 6.04 | 7.54 | 26.00 | 40.61 |
| **14** | -8.23 | 15.29 | 8.57 | 16.25 | -8.26 | 11.06 | 25.16 | 8.43 | 34.44 | 13.84 | 32.75 | 9.84 | 5.94 | 3.06 | 0.29 | 0.09 | 13.80 | 11.82 |
| **15** | -7.33 | 9.45 | 12.94 | 6.80 | -7.12 | -3.30 | -13.16 | 1.33 | 14.82 | -2.31 | -13.98 | -14.30 | -25.18 | 25.74 | 0.78 | -11.65 | -44.59 | -23.86 |
| **16** | -45.52 | 16.85 | 47.96 | -41.63 | -46.29 | -66.91 | -15.22 | 39.03 | 18.66 | -26.97 | -13.81 | -49.37 | 3.94 | 42.36 | 16.58 | -38.65 | 17.94 | -56.04 |
| **17** | 3.28 | 19.97 | 2.91 | 33.39 | 1.37 | 15.85 | -1.19 | 6.48 | 5.32 | -3.13 | 0.33 | -6.12 | 3.18 | 8.78 | 4.86 | 3.32 | 2.54 | 9.62 |
| **18** | 7.58 | 11.77 | 9.76 | 4.08 | 9.05 | -43.89 | -0.25 | 3.10 | 3.39 | -1.03 | 0.40 | -59.00 | 4.66 | 25.27 | 7.44 | 5.13 | 4.38 | 5.41 |
| **19** | -8.94 | 7.61 | 0.96 | 2.36 | -9.22 | -4.32 | 10.33 | 31.75 | 11.62 | 26.19 | 4.06 | 0.63 | -3.77 | 34.04 | 7.50 | 37.74 | -15.87 | 52.38 |
| **20** | 7.09 | 10.06 | 7.53 | 10.48 | 7.06 | 13.48 | 14.97 | 15.64 | 28.95 | 3.52 | 16.91 | 9.84 | -12.05 | 23.05 | 9.54 | -1.30 | -25.21 | -15.15 |

**Table C.** Symmetry index (%) of CoP parameters symmetric index for each neurological healthy individual in bipedal, tandem and unipedal conditions. Total disp. – total displacement of sway; vel. of sway – mean velocity of sway; AP – anterior-posterior; ML – mediolateral.

|  |  | **BIPEDAL CONDITION** | | | | |  | **TANDEM CONDITION** | | | | |  | **UNIPEDAL CONDITION** | | | | |
| --- | --- | --- | --- | --- | --- | --- | --- | --- | --- | --- | --- | --- | --- | --- | --- | --- | --- | --- |
| **Part.** | **total disp.** | **AP vel. of sway** | **ML vel. of sway** | **AP RMS** | **ML RMS** | **area of sway** | **total disp.** | **AP vel. of sway** | **ML vel. of sway** | **AP RMS** | **ML RMS** | **area of sway** | **total disp.** | **AP vel. of sway** | **ML vel. of sway** | **AP RMS** | **ML RMS** | **area of sway** |
| **1** | -12.96 | 17.38 | 5.25 | 17.92 | -12.46 | -1.01 | 10.07 | 14.28 | 10.75 | 13.93 | 9.76 | 31.39 | -30.63 | 1.45 | 4.27 | -21.40 | -34.86 | -40.33 |
| **2** | -0.59 | 3.47 | 2.76 | -26.12 | -0.03 | -25.12 | -4.52 | 2.96 | 1.16 | -11.05 | -1.86 | -47.62 | 1.73 | 1.64 | 5.32 | -0.76 | 1.72 | 1.15 |
| **3** | -8.84 | 15.36 | 9.98 | -29.87 | -8.07 | -23.23 | 0.13 | 15.46 | 1.18 | -7.59 | 6.66 | -20.80 | 3.96 | 7.04 | 13.60 | -0.49 | 8.16 | 14.04 |
| **4** | 19.48 | 14.25 | 20.23 | -15.52 | 21.84 | 19.89 | -11.43 | 20.19 | 6.34 | -7.56 | -16.95 | 2.58 | 22.01 | 27.26 | 32.82 | 23.51 | 18.86 | 50.55 |
| **5** | -7.07 | 17.13 | 12.82 | -24.62 | -8.16 | -18.46 | -30.52 | 24.65 | 36.18 | -21.77 | -36.60 | -43.88 | -7.79 | 5.35 | 11.99 | -5.38 | -6.96 | -11.68 |
| **6** | -13.41 | 6.56 | 24.25 | -13.89 | -13.22 | -28.39 | 11.71 | 32.34 | 48.96 | -21.64 | 37.79 | 35.17 | 28.00 | 25.90 | 2.57 | 18.42 | 35.43 | 58.13 |
| **7** | 15.08 | 2.69 | 17.09 | 16.99 | 14.72 | 20.18 | -11.05 | 24.37 | 3.16 | 10.22 | -16.64 | -5.65 | 16.24 | 59.93 | 25.40 | 45.33 | -5.74 | 43.94 |
| **8** | -13.30 | 3.51 | 9.79 | 5.77 | -11.83 | -15.96 | 4.10 | 8.17 | 22.52 | 9.79 | 1.42 | -6.07 | 13.09 | 11.39 | 4.14 | 19.48 | 8.21 | 27.50 |
| **9** | 14.25 | 24.69 | 23.06 | 35.44 | 13.83 | 13.17 | 28.81 | 21.39 | 28.99 | 18.37 | 30.81 | 22.34 | 0.07 | 6.85 | 6.86 | -10.73 | 7.33 | -3.98 |
| **10** | 3.99 | 10.52 | 7.83 | 11.09 | -0.06 | 25.25 | -13.92 | 1.38 | 1.68 | -1.67 | -22.01 | -7.76 | 2.31 | 1.98 | 23.08 | 4.27 | 0.80 | 7.57 |
| **11** | -33.53 | 14.54 | 46.40 | 0.85 | -34.31 | -43.90 | -9.62 | 2.88 | 1.19 | -10.55 | -13.87 | -23.31 | 8.24 | 9.88 | 18.78 | 10.19 | 3.64 | 13.49 |
| **12** | 5.91 | 2.31 | 0.79 | 38.58 | 4.23 | -6.73 | -4.91 | 15.94 | 10.16 | -11.66 | -4.77 | -8.41 | -4.59 | 2.54 | 1.12 | 6.13 | -14.89 | -5.43 |
| **13** | 15.96 | 5.48 | 6.13 | -9.96 | 15.69 | 12.76 | -4.41 | 14.08 | 27.59 | 19.75 | -19.67 | 13.47 | 0.04 | 14.08 | 9.95 | 3.26 | 0.01 | 3.22 |
| **14** | -10.33 | 1.32 | 13.15 | -2.98 | -10.36 | 3.88 | -8.96 | 4.99 | 0.11 | -2.50 | -9.90 | -26.77 | 9.47 | 27.35 | 15.05 | -7.03 | 16.77 | 0.76 |
| **15** | 11.11 | 0.54 | 12.27 | 18.94 | 11.28 | 3.31 | 10.83 | 18.03 | 2.00 | 17.99 | 7.21 | 18.00 | -2.25 | 2.35 | 10.78 | -6.37 | -0.11 | -9.62 |
| **16** | -3.23 | 4.16 | 5.47 | 2.65 | -3.87 | 0.25 | -12.44 | 8.33 | 8.83 | -9.48 | -12.09 | -9.85 | -0.41 | 29.14 | 31.41 | 0.79 | -3.76 | -7.86 |
| **17** | -5.93 | 2.65 | 2.99 | -20.97 | -4.42 | -11.91 | 4.91 | 1.95 | 4.53 | -8.62 | 5.36 | -7.61 | -12.12 | 2.39 | 5.83 | -15.59 | -11.26 | -20.15 |
| **18** | -23.35 | 7.72 | 25.30 | -1.74 | -23.51 | -31.74 | -14.76 | 11.48 | 20.07 | -9.57 | -18.91 | -17.20 | -4.65 | 34.72 | 2.28 | 35.66 | -34.26 | 8.45 |
| **19** | -1.96 | 19.58 | 4.55 | -26.72 | 3.22 | -3.75 | 14.47 | 4.06 | 7.47 | 5.55 | 12.85 | -8.89 | 13.69 | 27.80 | 22.31 | 24.20 | 3.87 | 44.92 |
| **20** | 0.46 | 4.70 | 1.94 | 18.30 | 0.11 | 1.41 | 3.58 | 8.92 | 3.23 | 12.31 | 0.26 | -16.89 | 18.11 | 44.23 | 30.10 | 32.65 | 8.38 | 36.15 |

**Table D.** Mean raw data of least affected limb for each patient with PD in bipedal, tandem and unipedal conditions. Total disp. – total displacement of sway; vel. of sway – mean velocity of sway; AP – anterior-posterior; ML – mediolateral.

|  | **BIPEDAL CONDITION** | | | | | | **TANDEM CONDITION** | | | | | | **UNIPEDAL CONDITION** | | | | | |
| --- | --- | --- | --- | --- | --- | --- | --- | --- | --- | --- | --- | --- | --- | --- | --- | --- | --- | --- |
| **Part.** | **total disp. (cm)** | **AP vel. of sway (cm/s)** | **ML vel. of sway (cm/s)** | **AP RMS** | **ML RMS** | **area of sway (cm^2^)** | **total disp. (cm)** | **AP vel. of sway (cm/s)** | **ML vel. of sway (cm/s)** | **AP RMS** | **ML RMS** | **area of sway (cm^2^)** | **total disp. (cm)** | **AP vel. of sway (cm/s)** | **ML vel. of sway (cm/s)** | **AP RMS** | **ML RMS** | **area of sway (cm^2^)** |
| **1** | 792.43 | 0.12 | 0.94 | 0.03 | 0.25 | 0.13 | 2468.30 | 1.35 | 2.81 | 0.35 | 0.70 | 0.75 | 1687.78 | 0.55 | 2.69 | 0.11 | 0.51 | 0.92 |
| **2** | 986.56 | 0.13 | 0.89 | 0.03 | 0.29 | 0.06 | 1840.35 | 1.95 | 4.13 | 0.29 | 0.49 | 0.45 | 1716.16 | 2.28 | 1.69 | 0.42 | 0.26 | 1.84 |
| **3** | 733.91 | 0.10 | 0.47 | 0.03 | 0.22 | 0.10 | 1181.22 | 0.27 | 0.87 | 0.14 | 0.33 | 0.23 | 1294.51 | 0.52 | 0.68 | 0.22 | 0.32 | 0.79 |
| **4** | 515.94 | 0.08 | 0.40 | 0.01 | 0.16 | 0.03 | 1399.92 | 0.40 | 1.36 | 0.13 | 0.41 | 0.27 | 1718.15 | 1.86 | 1.48 | 0.36 | 0.32 | 2.19 |
| **5** | 1138.51 | 0.12 | 0.71 | 0.05 | 0.34 | 0.24 | 1947.32 | 0.74 | 1.47 | 0.38 | 0.43 | 0.52 | 1669.95 | 0.57 | 1.39 | 0.20 | 0.46 | 0.90 |
| **6** | 1560.19 | 0.16 | 1.47 | 0.04 | 0.48 | 0.18 | 2485.27 | 1.28 | 2.19 | 0.40 | 0.68 | 0.78 | 2005.15 | 1.76 | 1.96 | 0.41 | 0.41 | 2.13 |
| **7** | 1176.06 | 0.13 | 0.69 | 0.03 | 0.37 | 0.17 | 1852.37 | 1.20 | 1.09 | 0.44 | 0.38 | 0.26 | 2657.36 | 3.09 | 1.99 | 0.66 | 0.43 | 3.39 |
| **8** | 696.54 | 0.10 | 0.41 | 0.02 | 0.23 | 0.06 | 1520.05 | 0.39 | 1.11 | 0.16 | 0.46 | 0.27 | 2462.07 | 1.50 | 1.50 | 0.50 | 0.49 | 4.69 |
| **9** | 508.54 | 0.10 | 0.36 | 0.06 | 0.12 | 0.04 | 1537.13 | 0.99 | 1.26 | 0.27 | 0.39 | 0.50 | 2540.11 | 2.01 | 2.14 | 0.46 | 0.57 | 4.81 |
| **10** | 910.80 | 0.14 | 1.21 | 0.03 | 0.28 | 0.08 | 1852.41 | 0.87 | 3.79 | 0.15 | 0.54 | 0.40 | 1443.71 | 0.65 | 2.41 | 0.15 | 0.42 | 0.60 |
| **11** | 821.08 | 0.44 | 1.68 | 0.03 | 0.25 | 0.09 | 2836.88 | 0.92 | 2.01 | 0.36 | 0.89 | 1.12 | 2470.95 | 0.85 | 1.58 | 0.35 | 0.71 | 3.66 |
| **12** | 643.91 | 0.12 | 0.50 | 0.03 | 0.20 | 0.04 | 2293.83 | 1.82 | 1.30 | 0.50 | 0.50 | 0.64 | 1931.02 | 1.95 | 1.09 | 0.39 | 0.38 | 2.77 |
| **13** | 564.05 | 0.22 | 0.40 | 0.05 | 0.17 | 0.16 | 1388.60 | 0.68 | 0.80 | 0.24 | 0.35 | 0.54 | 1394.52 | 0.56 | 0.98 | 0.15 | 0.40 | 1.03 |
| **14** | 871.31 | 0.11 | 0.42 | 0.02 | 0.27 | 0.07 | 2266.25 | 1.05 | 1.70 | 0.38 | 0.60 | 0.81 | 2524.69 | 2.06 | 1.45 | 0.49 | 0.53 | 4.70 |
| **15** | 440.43 | 0.09 | 0.25 | 0.02 | 0.14 | 0.04 | 1806.82 | 0.44 | 0.78 | 0.28 | 0.52 | 0.24 | 915.02 | 0.64 | 0.51 | 0.24 | 0.13 | 0.48 |
| **16** | 500.10 | 0.09 | 0.22 | 0.02 | 0.15 | 0.03 | 1223.91 | 0.27 | 1.02 | 0.11 | 0.38 | 0.14 | 1147.97 | 0.16 | 0.70 | 0.09 | 0.35 | 0.16 |
| **17** | 926.70 | 0.15 | 0.44 | 0.08 | 0.26 | 0.07 | 1900.93 | 0.93 | 1.29 | 0.35 | 0.49 | 0.21 | 1770.38 | 1.57 | 1.71 | 0.32 | 0.40 | 2.03 |
| **18** | 937.31 | 0.18 | 0.74 | 0.06 | 0.28 | 0.07 | 1623.67 | 0.93 | 1.73 | 0.30 | 0.40 | 0.18 | 1424.49 | 1.68 | 1.50 | 0.28 | 0.29 | 1.18 |
| **19** | 1129.42 | 0.12 | 0.74 | 0.02 | 0.36 | 0.16 | 1877.67 | 1.27 | 1.86 | 0.32 | 0.47 | 0.33 | 1628.53 | 1.27 | 1.31 | 0.28 | 0.38 | 1.62 |
| **20** | 567.82 | 0.13 | 0.47 | 0.03 | 0.17 | 0.05 | 1504.01 | 0.88 | 2.11 | 0.18 | 0.43 | 0.33 | 1005.02 | 1.39 | 1.13 | 0.22 | 0.19 | 0.63 |

**Table E.** Mean raw data of most affected limb for each patient with PD in bipedal, tandem and unipedal conditions. Total disp. – total displacement of sway; vel. of sway – mean velocity of sway; AP – anterior-posterior; ML – mediolateral.

|  | **BIPEDAL CONDITION** | | | | | | **TANDEM CONDITION** | | | | | | **UNIPEDAL CONDITION** | | | | | |
| --- | --- | --- | --- | --- | --- | --- | --- | --- | --- | --- | --- | --- | --- | --- | --- | --- | --- | --- |
| **Part.** | **total disp. (cm)** | **AP vel. of sway (cm/s)** | **ML vel. of sway (cm/s)** | **AP RMS** | **ML RMS** | **area of sway (cm^2^)** | **total disp. (cm)** | **AP vel. of sway (cm/s)** | **ML vel. of sway (cm/s)** | **AP RMS** | **ML RMS** | **area of sway (cm^2^)** | **total disp. (cm)** | **AP vel. of sway (cm/s)** | **ML vel. of sway (cm/s)** | **AP RMS** | **ML RMS** | **area of sway (cm^2^)** |
| **1** | 861.03 | 0.16 | 0.83 | 0.04 | 0.26 | 0.10 | 2398.21 | 2.30 | 2.97 | 0.47 | 0.59 | 0.29 | 2069.37 | 3.00 | 1.94 | 0.46 | 0.37 | 3.04 |
| **2** | 692.90 | 0.10 | 0.57 | 0.02 | 0.22 | 0.08 | 1812.89 | 2.03 | 3.71 | 0.30 | 0.48 | 0.45 | 1271.48 | 0.65 | 1.14 | 0.21 | 0.32 | 0.78 |
| **3** | 960.54 | 0.12 | 0.60 | 0.04 | 0.30 | 0.16 | 1274.81 | 0.59 | 0.84 | 0.21 | 0.32 | 0.50 | 1380.57 | 1.28 | 0.86 | 0.32 | 0.23 | 1.36 |
| **4** | 416.05 | 0.08 | 0.23 | 0.01 | 0.12 | 0.03 | 1303.62 | 0.44 | 1.28 | 0.13 | 0.38 | 0.31 | 2125.26 | 2.00 | 2.07 | 0.38 | 0.49 | 3.47 |
| **5** | 1314.60 | 0.12 | 0.85 | 0.05 | 0.39 | 0.25 | 2037.16 | 1.04 | 1.65 | 0.33 | 0.55 | 0.40 | 1241.15 | 0.80 | 1.11 | 0.21 | 0.30 | 0.95 |
| **6** | 636.49 | 0.09 | 0.42 | 0.02 | 0.19 | 0.05 | 961.62 | 0.46 | 0.83 | 0.15 | 0.24 | 0.21 | 2314.62 | 1.32 | 1.56 | 0.45 | 0.57 | 1.46 |
| **7** | 1118.40 | 0.11 | 0.51 | 0.05 | 0.35 | 0.16 | 2510.36 | 1.69 | 1.89 | 0.54 | 0.57 | 0.58 | 2169.21 | 1.28 | 2.79 | 0.33 | 0.56 | 1.83 |
| **8** | 950.41 | 0.14 | 0.54 | 0.04 | 0.30 | 0.08 | 2743.12 | 1.28 | 1.83 | 0.53 | 0.68 | 0.32 | 1208.18 | 0.82 | 0.75 | 0.27 | 0.21 | 1.07 |
| **9** | 588.41 | 0.09 | 0.45 | 0.06 | 0.13 | 0.03 | 1427.04 | 0.87 | 1.20 | 0.26 | 0.37 | 0.30 | 2279.45 | 1.85 | 1.90 | 0.44 | 0.46 | 3.78 |
| **10** | 1180.00 | 0.16 | 1.58 | 0.03 | 0.37 | 0.16 | 1676.17 | 1.00 | 2.72 | 0.20 | 0.48 | 0.50 | 1579.85 | 1.47 | 2.70 | 0.23 | 0.41 | 1.12 |
| **11** | 875.08 | 0.43 | 1.77 | 0.03 | 0.27 | 0.11 | 2548.98 | 0.95 | 1.93 | 0.33 | 0.72 | 0.85 | 2235.65 | 1.07 | 1.25 | 0.34 | 0.52 | 3.11 |
| **12** | 477.53 | 0.11 | 0.39 | 0.02 | 0.15 | 0.03 | 2004.36 | 0.99 | 1.11 | 0.41 | 0.51 | 0.78 | 1577.00 | 0.97 | 1.43 | 0.24 | 0.43 | 1.87 |
| **13** | 1039.97 | 0.16 | 0.87 | 0.04 | 0.33 | 0.24 | 1969.78 | 0.87 | 1.39 | 0.34 | 0.50 | 0.53 | 945.23 | 0.43 | 0.87 | 0.13 | 0.23 | 0.43 |
| **14** | 1027.52 | 0.08 | 0.50 | 0.01 | 0.31 | 0.06 | 1355.09 | 0.89 | 0.83 | 0.29 | 0.30 | 0.66 | 2241.51 | 1.93 | 1.44 | 0.49 | 0.40 | 3.71 |
| **15** | 510.13 | 0.11 | 0.33 | 0.01 | 0.16 | 0.04 | 2354.28 | 0.46 | 1.05 | 0.29 | 0.69 | 0.32 | 1530.98 | 0.38 | 0.51 | 0.30 | 0.35 | 0.78 |
| **16** | 1335.65 | 0.12 | 0.64 | 0.04 | 0.42 | 0.15 | 1663.31 | 0.60 | 1.50 | 0.20 | 0.50 | 0.43 | 1060.85 | 0.61 | 0.97 | 0.20 | 0.24 | 0.57 |
| **17** | 867.91 | 0.10 | 0.47 | 0.04 | 0.25 | 0.05 | 1946.55 | 1.06 | 1.43 | 0.38 | 0.49 | 0.24 | 1661.29 | 1.87 | 1.55 | 0.30 | 0.38 | 1.67 |
| **18** | 805.23 | 0.23 | 0.90 | 0.05 | 0.24 | 0.18 | 1631.78 | 0.99 | 1.62 | 0.30 | 0.40 | 0.69 | 1297.53 | 1.00 | 1.29 | 0.25 | 0.27 | 1.06 |
| **19** | 1351.17 | 0.10 | 0.75 | 0.02 | 0.43 | 0.18 | 1526.20 | 0.66 | 1.47 | 0.19 | 0.44 | 0.33 | 1756.28 | 0.43 | 1.53 | 0.13 | 0.53 | 0.51 |
| **20** | 492.67 | 0.10 | 0.40 | 0.02 | 0.15 | 0.04 | 1112.38 | 0.64 | 1.16 | 0.16 | 0.30 | 0.27 | 1280.39 | 0.87 | 0.93 | 0.22 | 0.31 | 0.86 |

**Table F.** Mean raw data of dominant limb for each neurological healthy individual in bipedal, tandem and unipedal conditions. Total disp. – total displacement of sway; vel. of sway – mean velocity of sway; AP – anterior-posterior; ML – mediolateral.

|  | **BIPEDAL CONDITION** | | | | | | **TANDEM CONDITION** | | | | | | **UNIPEDAL CONDITION** | | | | | |
| --- | --- | --- | --- | --- | --- | --- | --- | --- | --- | --- | --- | --- | --- | --- | --- | --- | --- | --- |
| **Part.** | **total disp. (cm)** | **AP vel. of sway (cm/s)** | **ML vel. of sway (cm/s)** | **AP RMS** | **ML RMS** | **area of sway (cm^2^)** | **total disp. (cm)** | **AP vel. of sway (cm/s)** | **ML vel. of sway (cm/s)** | **AP RMS** | **ML RMS** | **area of sway (cm^2^)** | **total disp. (cm)** | **AP vel. of sway (cm/s)** | **ML vel. of sway (cm/s)** | **AP RMS** | **ML RMS** | **area of sway (cm^2^)** |
| **1** | 815.68 | 0.14 | 0.48 | 0.02 | 0.24 | 0.05 | 2123.74 | 0.86 | 1.85 | 0.26 | 0.58 | 0.38 | 2120.92 | 0.83 | 1.02 | 0.33 | 0.53 | 2.46 |
| **2** | 810.68 | 0.14 | 0.70 | 0.04 | 0.25 | 0.17 | 1403.76 | 0.95 | 1.89 | 0.31 | 0.30 | 0.84 | 2933.77 | 3.57 | 2.85 | 0.62 | 0.56 | 6.45 |
| **3** | 834.57 | 0.11 | 0.51 | 0.05 | 0.25 | 0.10 | 1624.92 | 0.95 | 1.26 | 0.37 | 0.35 | 0.64 | 2558.12 | 2.54 | 1.93 | 0.58 | 0.46 | 4.31 |
| **4** | 905.18 | 0.16 | 0.78 | 0.05 | 0.28 | 0.11 | 2743.36 | 0.36 | 2.84 | 0.19 | 0.87 | 0.54 | 1089.62 | 0.99 | 1.23 | 0.18 | 0.26 | 0.69 |
| **5** | 624.47 | 0.11 | 0.45 | 0.03 | 0.19 | 0.06 | 1526.02 | 0.58 | 0.75 | 0.22 | 0.43 | 0.82 | 2584.28 | 2.58 | 1.99 | 0.50 | 0.52 | 4.81 |
| **6** | 714.41 | 0.20 | 0.22 | 0.04 | 0.22 | 0.05 | 655.61 | 0.51 | 1.41 | 0.15 | 0.11 | 0.09 | 1704.08 | 2.29 | 2.12 | 0.41 | 0.31 | 1.72 |
| **7** | 405.12 | 0.15 | 0.42 | 0.02 | 0.13 | 0.03 | 2297.89 | 1.27 | 2.13 | 0.23 | 0.73 | 0.63 | 870.93 | 1.00 | 0.89 | 0.10 | 0.24 | 0.43 |
| **8** | 862.87 | 0.09 | 0.82 | 0.01 | 0.27 | 0.07 | 1711.50 | 1.30 | 0.92 | 0.35 | 0.35 | 0.53 | 1947.76 | 3.04 | 2.94 | 0.36 | 0.42 | 2.82 |
| **9** | 593.09 | 0.20 | 0.88 | 0.02 | 0.19 | 0.06 | 1069.29 | 0.99 | 2.54 | 0.14 | 0.30 | 0.49 | 2132.53 | 2.12 | 2.32 | 0.47 | 0.40 | 3.50 |
| **10** | 1394.25 | 0.15 | 0.73 | 0.06 | 0.44 | 0.10 | 2775.37 | 1.40 | 2.67 | 0.36 | 0.86 | 1.13 | 2057.74 | 1.87 | 2.41 | 0.41 | 0.42 | 2.97 |
| **11** | 680.88 | 0.11 | 0.29 | 0.02 | 0.21 | 0.06 | 818.27 | 0.42 | 0.88 | 0.10 | 0.25 | 0.14 | 1551.09 | 1.90 | 1.61 | 0.31 | 0.32 | 1.83 |
| **12** | 539.24 | 0.11 | 0.28 | 0.02 | 0.17 | 0.04 | 1467.39 | 0.39 | 1.02 | 0.20 | 0.44 | 0.23 | 2558.45 | 1.77 | 1.43 | 0.44 | 0.56 | 4.25 |
| **13** | 610.41 | 0.10 | 0.62 | 0.03 | 0.19 | 0.08 | 1442.34 | 0.83 | 1.10 | 0.18 | 0.43 | 0.66 | 2783.69 | 2.54 | 2.43 | 0.56 | 0.52 | 5.51 |
| **14** | 807.25 | 0.15 | 0.63 | 0.04 | 0.26 | 0.09 | 2380.85 | 1.03 | 2.31 | 0.26 | 0.68 | 0.55 | 1360.72 | 0.60 | 1.28 | 0.20 | 0.34 | 1.15 |
| **15** | 655.90 | 0.15 | 0.56 | 0.02 | 0.20 | 0.07 | 1612.63 | 1.02 | 1.84 | 0.21 | 0.48 | 0.45 | 2725.13 | 2.99 | 3.26 | 0.56 | 0.55 | 5.68 |
| **16** | 932.51 | 0.86 | 0.70 | 0.05 | 0.28 | 0.19 | 2126.04 | 1.67 | 3.35 | 0.17 | 0.64 | 0.59 | 1815.09 | 1.90 | 1.86 | 0.37 | 0.36 | 2.46 |
| **17** | 1120.67 | 0.53 | 0.60 | 0.06 | 0.33 | 0.21 | 2015.12 | 0.76 | 2.13 | 0.27 | 0.58 | 0.47 | 1631.35 | 0.78 | 1.06 | 0.27 | 0.39 | 1.67 |
| **18** | 764.50 | 0.59 | 0.57 | 0.05 | 0.22 | 0.16 | 1926.17 | 0.83 | 1.64 | 0.24 | 0.57 | 0.41 | 1537.92 | 1.42 | 0.98 | 0.17 | 0.42 | 1.21 |
| **19** | 1083.41 | 0.91 | 0.92 | 0.11 | 0.32 | 0.10 | 2278.56 | 1.67 | 3.17 | 0.40 | 0.62 | 0.58 | 1553.83 | 1.27 | 1.58 | 0.23 | 0.40 | 1.15 |
| **20** | 921.94 | 0.56 | 0.86 | 0.04 | 0.28 | 0.18 | 1891.12 | 1.21 | 2.05 | 0.25 | 0.53 | 0.36 | 1438.24 | 1.99 | 2.17 | 0.22 | 0.36 | 1.36 |

**Table G.** Mean raw data of non-dominant limb for each neurological healthy individual in bipedal, tandem and unipedal conditions. Total disp. – total displacement of sway; vel. of sway – mean velocity of sway; AP – anterior-posterior; ML – mediolateral.

|  | **BIPEDAL CONDITION** | | | | | | **TANDEM CONDITION** | | | | | | **UNIPEDAL CONDITION** | | | | | |
| --- | --- | --- | --- | --- | --- | --- | --- | --- | --- | --- | --- | --- | --- | --- | --- | --- | --- | --- |
| **Part.** | **total disp. (cm)** | **AP vel. of sway (cm/s)** | **ML vel. of sway (cm/s)** | **AP RMS** | **ML RMS** | **area of sway (cm^2^)** | **total disp. (cm)** | **AP vel. of sway (cm/s)** | **ML vel. of sway (cm/s)** | **AP RMS** | **ML RMS** | **area of sway (cm^2^)** | **total disp. (cm)** | **AP vel. of sway (cm/s)** | **ML vel. of sway (cm/s)** | **AP RMS** | **ML RMS** | **area of sway (cm^2^)** |
| **1** | 628.47 | 0.10 | 0.53 | 0.03 | 0.19 | 0.05 | 2599.42 | 0.65 | 1.49 | 0.35 | 0.70 | 0.73 | 1126.30 | 0.81 | 0.93 | 0.22 | 0.26 | 1.05 |
| **2** | 801.19 | 0.13 | 0.67 | 0.02 | 0.25 | 0.10 | 1282.36 | 0.90 | 1.84 | 0.25 | 0.29 | 0.30 | 3037.08 | 3.45 | 2.56 | 0.61 | 0.58 | 6.60 |
| **3** | 699.06 | 0.14 | 0.63 | 0.02 | 0.21 | 0.06 | 1629.29 | 1.29 | 1.23 | 0.32 | 0.40 | 0.42 | 2769.13 | 2.21 | 1.47 | 0.58 | 0.54 | 5.71 |
| **4** | 1343.04 | 0.12 | 0.52 | 0.04 | 0.44 | 0.17 | 2180.68 | 0.55 | 2.50 | 0.16 | 0.62 | 0.57 | 1704.47 | 0.56 | 0.62 | 0.30 | 0.38 | 2.11 |
| **5** | 541.97 | 0.15 | 0.59 | 0.02 | 0.16 | 0.04 | 812.27 | 0.96 | 1.60 | 0.14 | 0.20 | 0.32 | 2210.58 | 2.88 | 2.53 | 0.45 | 0.45 | 3.80 |
| **6** | 545.47 | 0.23 | 0.37 | 0.03 | 0.17 | 0.03 | 829.53 | 0.26 | 0.48 | 0.10 | 0.24 | 0.19 | 3029.43 | 1.35 | 2.02 | 0.59 | 0.66 | 6.51 |
| **7** | 548.96 | 0.14 | 0.30 | 0.03 | 0.17 | 0.05 | 1840.65 | 0.77 | 2.00 | 0.28 | 0.52 | 0.56 | 1208.60 | 0.25 | 0.53 | 0.27 | 0.21 | 1.12 |
| **8** | 660.35 | 0.08 | 1.00 | 0.02 | 0.21 | 0.05 | 1857.80 | 1.53 | 1.46 | 0.43 | 0.36 | 0.47 | 2534.53 | 2.42 | 3.19 | 0.54 | 0.49 | 4.96 |
| **9** | 790.28 | 0.12 | 0.55 | 0.04 | 0.25 | 0.08 | 1934.92 | 0.64 | 1.40 | 0.21 | 0.56 | 0.77 | 2135.53 | 2.44 | 2.02 | 0.38 | 0.46 | 3.24 |
| **10** | 1510.19 | 0.12 | 0.85 | 0.07 | 0.44 | 0.16 | 2097.02 | 1.37 | 2.58 | 0.34 | 0.55 | 0.97 | 2155.02 | 1.79 | 1.50 | 0.44 | 0.43 | 3.46 |
| **11** | 338.90 | 0.15 | 0.80 | 0.02 | 0.10 | 0.02 | 674.63 | 0.39 | 0.90 | 0.08 | 0.19 | 0.08 | 1829.78 | 1.56 | 1.10 | 0.38 | 0.34 | 2.40 |
| **12** | 607.03 | 0.11 | 0.28 | 0.04 | 0.19 | 0.04 | 1329.95 | 0.54 | 1.25 | 0.16 | 0.40 | 0.19 | 2333.91 | 1.87 | 1.46 | 0.50 | 0.41 | 3.81 |
| **13** | 842.19 | 0.11 | 0.55 | 0.02 | 0.26 | 0.10 | 1320.41 | 0.63 | 1.93 | 0.26 | 0.29 | 0.87 | 2785.71 | 3.37 | 2.97 | 0.60 | 0.52 | 5.87 |
| **14** | 656.06 | 0.15 | 0.81 | 0.04 | 0.21 | 0.10 | 1989.31 | 0.93 | 2.31 | 0.25 | 0.55 | 0.32 | 1645.28 | 1.05 | 1.74 | 0.17 | 0.47 | 1.16 |
| **15** | 819.91 | 0.15 | 0.44 | 0.03 | 0.25 | 0.07 | 2004.20 | 0.71 | 1.77 | 0.30 | 0.55 | 0.65 | 2605.34 | 3.13 | 2.63 | 0.49 | 0.55 | 4.68 |
| **16** | 874.19 | 0.93 | 0.79 | 0.05 | 0.26 | 0.19 | 1655.67 | 1.42 | 2.81 | 0.14 | 0.50 | 0.49 | 1800.15 | 1.04 | 0.97 | 0.38 | 0.33 | 2.10 |
| **17** | 995.18 | 0.56 | 0.56 | 0.04 | 0.30 | 0.16 | 2223.23 | 0.79 | 1.94 | 0.22 | 0.65 | 0.41 | 1278.69 | 0.82 | 0.94 | 0.20 | 0.31 | 1.11 |
| **18** | 475.01 | 0.69 | 0.95 | 0.05 | 0.14 | 0.08 | 1430.76 | 1.04 | 2.46 | 0.20 | 0.39 | 0.29 | 1401.29 | 0.69 | 0.94 | 0.36 | 0.20 | 1.44 |
| **19** | 1041.75 | 0.36 | 0.84 | 0.06 | 0.34 | 0.09 | 3049.65 | 1.54 | 2.73 | 0.44 | 0.80 | 0.49 | 2046.58 | 0.72 | 1.00 | 0.38 | 0.43 | 3.03 |
| **20** | 930.49 | 0.61 | 0.82 | 0.06 | 0.29 | 0.19 | 2031.46 | 1.01 | 2.19 | 0.32 | 0.53 | 0.26 | 2074.52 | 0.77 | 1.16 | 0.43 | 0.42 | 2.90 |
